# The role of p53 in male infertility

**DOI:** 10.3389/fendo.2024.1457985

**Published:** 2024-10-14

**Authors:** Jing Li, Xia Huang, Lei Luo, Jialin Sun, Qie Guo, Xue Yang, Chuanzhou Zhang, Beibei Ni

**Affiliations:** ^1^ Department of Pharmacy, The Affiliated Hospital of Qingdao University, Qingdao, China; ^2^ Department of Human Resource, The Affiliated Hospital of Qingdao University, Qingdao, China; ^3^ Department of Urology, The Affiliated Hospital of Qingdao University, Qingdao, China

**Keywords:** p53, male infertility, spermatogenesis, drug target, strategy

## Abstract

The tumor suppressor p53 is a transcription factor involved in a variety of crucial cellular functions, including cell cycle arrest, DNA repair and apoptosis. Still, a growing number of studies indicate that p53 plays multiple roles in spermatogenesis, as well as in the occurrence and development of male infertility. The representative functions of p53 in spermatogenesis include the proliferation of spermatogonial stem cells (SSCs), spermatogonial differentiation, spontaneous apoptosis, and DNA damage repair. p53 is involved in various male infertility-related diseases. Innovative therapeutic strategies targeting p53 have emerged in recent years. This review focuses on the role of p53 in spermatogenesis and male infertility and analyses the possible underlying mechanism involved. All these conclusions may provide a new perspective on drug intervention targeting p53 for male infertility treatment.

## 1 Introduction

Infertility is defined as the failure to conceive after 12 months or more of regular, unprotected sexual intercourse (1). Infertility is a major health problem and social challenge, particularly in an era of low fertility and an aging society (2). The incidence of infertility is estimated to be 12.6%-17.5% among reproductive-aged couples worldwide, and males are responsible for approximately half of all infertility problems (3). In recent years, the increasing prevalence of male infertility has attracted global attention. An analysis of the Global Burden of Disease (GBD) 2019 showed that the global prevalence of male infertility increased significantly, by 76.9%, from 1990 to 2019, along with a steady growth trend in the global burden of male infertility diseases (4). Multiple studies have shown worldwide decreases in sperm counts (5–7). A recent systematic review by Levine et al. (8) revealed that the total sperm count declined by 62.3% between 1973 and 2018, and this decrease has accelerated since 2000. Various causes involved in male infertility have been discussed, mainly including congenital factors (e.g., genetic abnormalities, congenital bilateral absence of the vas deferens, and cryptorchidism), acquired factors (e.g., varicocele, testicular torsion, and genital inflammation), and idiopathic risk factors (e.g., smoking, obesity, and aging) (9). Although significant progress has been made within the past few decades, a considerable proportion of cases of male infertility are diagnosed as idiopathic or unexplained, which means that the underlying mechanisms have not yet been revealed (10). There has also been little progress in drug therapy in the past decade (11). Although the use of assisted reproductive technology (ART) can bring dawn to infertile men, it is not always a panacea, and its safety has been questioned because of comorbidities in the offspring (12). Therefore, research on male infertility has become a global endeavor.

The tumor suppressor protein p53, encoded by *TP53* in human and by *Tp53* in mice, is a vital regulator of the cellular stress response and a crucial controller of cell fate (13). p53 contains five domains: (1) the transactivation domain (TAD), which is responsible for gene transcription; (2) the proline-rich domain (PRD), which is crucial for p53 stability; (3) the DNA-binding domain (DBD), which is required for DNA binding activity; (4) the tetramerization domain (TD), which is needed for tetramerization of p53; and (5) the C-terminal domain, which is responsible for regulating the binding activity between p53 and the DBD and is influenced by posttranslational modifications (PTMs) of p53 (14). Among these modifications, phosphorylation and acetylation stabilize p53 and increase transcriptional activation, while the effect of methylation on p53 transcriptional activity varies with lysine residues (15). Under physiological conditions, p53 is maintained at a low level mainly due to E3 ligase Mdm2-mediated ubiquitin-mediated degradation. Under stress conditions, p53 can be activated by PTMs or reduced degradation (16). As a transcription factor, p53 is involved in a variety of crucial cellular functions, including cell cycle arrest, DNA repair, apoptosis, autophagy, senescence, and oxidative stress (14).

Multiple studies have shown that p53 is closely related to many developmental disorders or diseases, such as stem cell differentiation, cancer, and cardiovascular and neurodegenerative diseases (17–19). In recent years, abundant evidence has indicated the crucial role of p53 in spermatogenesis and the pathogenesis of male infertility (20, 21). Excitingly, p53 has proven to be druggable, and efforts toward the development of p53-based therapy are emerging (22). Therefore, we summarized the roles of p53 in spermatogenesis and the mechanisms of p53 in male infertility diseases, which may provide new strategies for male infertility therapies.

## 2 p53 and spermatogenesis

**Figure 1:**
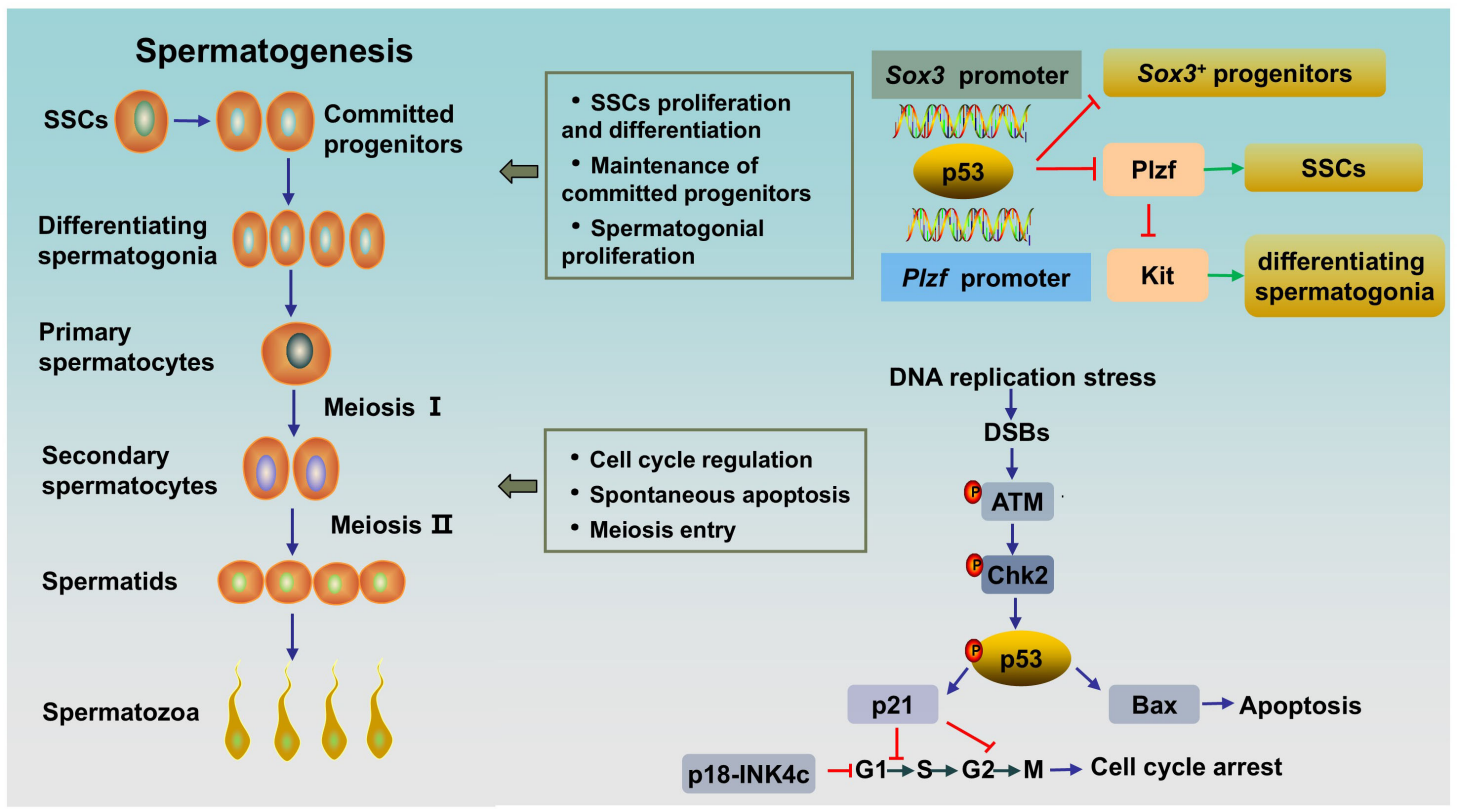
The role of p53 in rodent spermatogenesis. SSCs differentiate into progenitor cells, and the latter subsequently differentiate into differentiating spermatogonia. Differentiating spermatogonia differentiate into spermatocytes, and spermatocytes produce spermatids via meiotic division. Afterwards, round spermatids undergo maturation via spermiogenesis to form spermatozoa. Activated p53 can bind to the *Plzf* promoter and the *Sox3* promoter to negatively regulate Plzf and Sox3, leading to a reduction in the number of *Plzf+* SSCs and *Sox3+* progenitor spermatogonia. During normal spermatogenesis, ATM/p53/Bax pathway or ATM/p53/p21 pathway are activated in DNA-damaged germ cells to induce spontaneous apoptosis or cell cycle arrest, respectively. SSCs, spermatogonial stem cells; DSBs, DNA double-strand breaks.

Spermatogenesis is a unique and complex cell cycle process that is characterized by fine regulation to ensure high-fidelity transmission of genetic material (23). Three stages are involved in spermatogenesis (**Figure 1**): spermatogonial stem cells (SSCs) differentiate into spermatocytes via mitotic proliferation, spermatocytes transform into spermatids via meiotic division, and spermatids differentiate into spermatozoa (24). p53 appears to be indispensable for normal spermatogenesis, a process that includes a great deal of DNA replication and DNA packaging (25). Here, we clarified the role of p53 in spermatogenesis (**Figure 1**).

### 2.1 The role of p53 in spermatogonia

The maintenance of spermatogenesis throughout the male’s lifetime relies on SSCs, which possess the ability to self-renew and differentiate into committed spermatogonial progenitor cells (SPCs) (26). SSCs are undifferentiated spermatogonia and a heterogeneous population. In rodent testes, SSCs can be subdivided into A_single_ (A_s_), A_paired_ (A_pr_) and A_aligned_ (A_al_) spermatogonia and can be distinguished by several genes, including *Ngn3*, *Sox3*, *Gfrα1* and *Plzf* (27). *Gfrα1^+^* cells (mainly A_s_, A_pr_, and some A_al_) are stem cells that can transform into *Ngn3/Sox3^+^* (mainly A_al_ and some A_pr_) committed SPCs. The latter differentiate into differentiating spermatogonia (type A1-A4, intermediate spermatogonia, type B), thereby entering meiosis after undergoing rapid mitotic division (28). PLZF (A_s_, A_pr_, and A_al_), whose key function is to maintain an undifferentiated state by directly repressing the expression of *Kit*, a hallmark of spermatogonial differentiation, is crucial for the self-renewal of SSCs (29, 30). By contrast, human testes contain two types of distinct A spermatogonia, identified as A_dark_ and A_pale_, based on nuclear morphology. A_pale_ are characterized by self-renewal and regular proliferation, while A_dark_ are slow cycling. There are 5 transit amplifying divisions in humans form A_dark_/A_pale_ undifferentiated spermatogonia to terminally differentiated sperm, while 12 transit amplifying divisions exist in rodents from A_s_ spermatogonia to terminally differentiated sperm. However, the larger pool of stem/progenitor cells partly compensates for fewer transit-amplifying cell divisions in human testis. Markers of undifferentiated spermatogonia in human also include GFRa1 and PLZF. And cKIT is a marker of differentiated spermatogonia in human testes, too (31).

It has been proven that p53 has multiple effects on spermatogonia. First, p53 has been shown to participate in the regulation of SSC proliferation and spermatogonial differentiation by regulating PLZF. Increased p53 can bind to the *Plzf* promoter and negatively regulate *Plzf*, leading to a reduction in *Plzf* expression and the number of *Plzf^+^* SSCs in mouse testes, resulting in a decrease in male fertility (32). Interestingly, it has been reported that PLZF could also act as a transcription repressor to decrease p53 levels by inhibiting *TP53* transcription and promoting the ubiquitination and degradation of p53 in several cell lines (33). The interaction between the two proteins might indicate the fine regulation of p53 in spermatogenesis. Second, p53 plays an important role in the maintenance of SPCs. A study showed that *Csnk1a1*, the gene encoded Casein kinase I isoform alpha (CKI-α), knockout accelerated nuclear p53 protein expression by disrupting Mdm2-mediated p53 ubiquitination in mouse spermatogonia. Subsequently, activated p53 negatively regulates *Sox3* by binding to the *Sox3* promoter, resulting in decreased *Sox3^+^* progenitor spermatogonia, blocked meiosis, and ultimately male infertility. As expected, the p53 inhibitor pifithrin-α partially rescued spermatogenesis in CKI-α-null mice (34). Finally, p53 has been shown to play a role in spermatogonial proliferation and apoptosis. Germ cells expand through mitosis, and p53-mediated apoptosis maintains germline homeostasis. Increased production of differentiating type spermatogonia and decreased clearance of lethally damaged differentiating type spermatogonia can be observed in *Tp53* knockout C57BL/6 mouse testes, indicating the key role of p53 in the regulation of cell proliferation and apoptosis in normal spermatogonia (35). Mechanistically, accelerated p53 ubiquitination-mediated degradation and reduced p53 activity result in a decrease in target molecules cyclin-dependent kinase inhibitor p21 and apoptosis regulator Bax levels, and an increase in Cyclin-dependent kinase 1 (CDK1) level, thereby accelerating the G2/M phase transition, inhibiting apoptosis and promoting spermatogonial proliferation (36). However, inconsistent results exist. A study by Dai et al. (25) revealed a normal and unaffected undifferentiated spermatogonial population and SSC proliferation rate, accompanied by elevated spermatocyte death and testicular atrophy in *Tp53* knockout rats on a Sprague-Dawley background. This inconsistency may be related to the different genetic backgrounds of the animals. In addition, p53 and cyclin-dependent kinase 4 inhibitor C (p18-INK4c, encoded by *Cdkn2c* in mice) collaborate to control the time from the mitosis phase to meiosis in spermatogonia. p18-INK4c acts as a negative regulator of the cell cycle at the early G1 phase (37). A study has shown that *Cdkn2c*/*Tp53* double knockout mice displayed higher percentage of PCNA (a marker of proliferation expressed in S phase) -positive tubules compared with that in wild type and *Cdkn2c*-null mice, which indicated *Cdkn2c*/*Tp53* double knockout mice existed active proliferation and continuation of the mitosis phase in spermatogonia, and thus a delay in meiosis entry (20). Besides, in this study, *Cdkn2c* null mice showed a significantly increased sperm counts as compared to *Tp53* null or wild type, while *Cdkn2c*/*Tp53* double knockout mice had much lower sperm counts, which might attributed to the increased apoptosis induced by DNA damage secondary to prolongation of meiosis entry (20). Overall, p53 plays an important role in the differentiation, proliferation, apoptosis and cell cycle regulation of SSCs and spermatogonia.

### 2.2 The role of p53 in spermatocytes

A1-type spermatogonia are transformed into preleptotene spermatocytes after six divisions and subsequently enter meiosis (38). After meiotic prophase, a prolonged G2 phase that allows a series of chromosome events with active genetic recombination, primary spermatocytes undergo meiosis I to form secondary spermatocytes (39). Subsequently, secondary spermatocytes undergo meiosis II to produce spermatids. The meiosis process can be divided into the following steps: DNA replication, crossover recombination, reductional division and equational division (40). Notably, meiosis is accompanied by DNA replication, DNA double-strand breaks (DSBs) and DNA damage repair (41). Consequently, fine control to maintain genomic stability and prevent mutagenesis is needed to avoid meiotic defects.

As the guardian of the genome, p53 appears to be duty-bound to respond to DNA damage during spermatogenesis. In contrast to those in other tissues, p53 levels are significantly greater in the testis. The expression of p53 is observed in the spermatogonium stage, peaks in primary spermatocytes, and slightly decreases in the sperm stage, which is consistent with DNA damage and recombination repair in the meiotic process (42). The high level of p53 in primary spermatocytes emphasizes its role in DNA repair at low DNA damage-dependent premeiotic checkpoints (43). Indeed, failure to resolve DSBs in leptotene and zygotene spermatocytes in *Tp53* knockout mice prior to synapsis leads to increased spermatocyte death and testis atrophy (25). Ataxia-telangiectasia mutated protein (ATM) acts as a sensor of DSBs. As an upstream regulator, activated ATM stabilizes p53 through direct or indirect phosphorylation by activating checkpoint kinase 2 (CHK2) (44). The response of activated p53 to damaged DNA is bidirectional. On the one hand, for repairable DNA damage, activated p53 can initiate cell cycle arrest via the downstream target p21, which arrests the cell cycle in G1/S or G2/M, to provide time for correcting DNA damage to promote cell entry into the next normal cell cycle (45). On the other hand, for DNA damage beyond cell repair, p53 induces apoptosis by activating proapoptotic factors (e.g., Bax and Caspase3) and inhibiting the antiapoptotic factor B-cell lymphoma-2 (Bcl-2) (44, 46).

Previous studies have shown that the level of p53 is the key factor that determines whether a cell undergoes growth arrest or apoptosis; that is, low p53 levels lead to growth arrest, while increased p53 levels result in apoptosis (47). Thus, the level of p53 must be precisely regulated. The Cullin-RING E3 ubiquitin ligase (CRL) is an ubiquitin ligase complex that can recognize substrates and then ubiquitinate and degrade them (48). CRL4 is a member of CRL family, which consists of Cullin4, a RING finger and DNA damage binding protein 1 (DDB1)-Cullin4 associated substrate receptors (49). CRL4 is involved in regulating the level of p53 to determine whether p53 induces growth arrest during spermatogenesis (42). It has been reported that Cullin4 promotes p53 ubiquitin degradation, followed by p21 transcriptional inactivation (46). Furthermore, p21 is also a substrate of Cullin. Inactivating the CRL E3 ligase by MLN4924, an inhibitor of Cullin neddylation, results in p21 accumulation. Interestingly, Cullin4, p53 and p21 exhibit similar expression patterns and intensities during spermatogenesis, and they are all highly expressed in primary spermatocytes (42). These findings indicate the critical role of Cullin4 in maintaining p53 homeostasis in primary spermatocytes.

### 2.3 p53 and germ cell death in spermatogenesis

The apoptosis of germ cells is a normal phenomenon in spermatogenesis and has been widely observed in SSCs, spermatogonia, spermatocytes and spermatids. Approximately 75% of germ cells will undergo spontaneous apoptosis (35). Spontaneous apoptosis occurs in all types of human male germ cells (50). The physiological significance of spontaneous apoptosis is, on the one hand, maintaining the appropriate number of sperm cells to adapt to the limited support capacity of Sertoli cells and, on the other hand, eliminating abnormal germ cells (51). Indeed, p53-mediated germ cell apoptosis is a crucial quality control mechanism. Multiple studies have shown that p53 overexpression or underexpression can impair spermatogenesis (**Table 1**). p53 eliminates DNA-damaged germ cells during normal spermatogenesis by inducing apoptosis, especially during the progression of meiosis, as we described in Section 2.2. *Tp53* knockout (*Tp53*-/-) mice exhibit decreased spontaneous apoptosis and increased germ numbers, along with an increased percentage of defective sperm and decreased fertility (56). In addition to spontaneous apoptosis, spermatogenesis also involves programmed necroptosis (57). A study by Napoletano et al. (52) have shown that p53 is required to regulate programmed necrosis in spermatogonia during *Drosophila* spermatogenesis to control germ cell number under physiological conditions. Furthermore, blocking this process can lead to hyperplasia of the *Drosophila* testes. However, spontaneous germ cell necrosis takes place very sporadically in the testes of wild type or *Tp53-/-* mutant mice at 6-8 weeks of age (52).

**Table 1:** Several phenotypes associated with spermatogenesis and function caused by *Tp53* genomic knockout or overexpression.

Models	Fertility	Phenotype	Ref.
*Tp53* knockout mice of C57BL background	—	Reduction in mature motile spermatozoa	(43)
Transgenic mice with a C57BL/6 genetic background expressing the wild-type *Tp53*	1. Mice with a high or moderate level of transgene expression are subfertility. 2. Mice with a low level of transgene expression are fertility.	1. Mice with a high level of transgene expression exhibit testicular atrophy and a markedly reduction in the amount of mature spermatozoa. 2. Mice with a moderate level of transgene expression have no significant change in testis weights and spermatozoa production, while teratozoospermia is obvious. 3. Increased spermatozoa defects in mice with a low level of transgene expression	(47)
*Tp53* knockout mice of C57BL/6 background	—	1. Normal testicular morphology 2. Mature spermatozoa exist in seminiferous tubules	(52)
*Tp53* null mice of C57BL/6×129 background	1. Homozygous mice [129(-/-)] are infertility. 2. Heterozygous mice [129(+/-)] are fertility.	1. Homozygous mice [129(-/-)] exhibit the testicular giant-cell degenerative syndrome. 2. Heterozygous mice [129(+/-)] exhibit a normal testicular morphology.	(53)
*Tp53* knockout mice of C57BL/6×129 background	—	1. Increased testis weight and sperm concentration 2. Increased proportion of abnormal sperm	(54)
Adenovirus-mediated *Tp53* overexpression in testis of adult Sprague-Dawley rats	—	1. Decreased testicular weight 2. Deteriorative seminiferous tubules 3. Spermatocytes and spermatids disappeared gradually	(55)

—, No data.

In addition, p53-mediated cell death is important for the response to adverse external stimuli. After irradiation, germ cell death in wild-type rat testes increased. However, there was no significant difference in the germ cell apoptotic response between irradiated or unirradiated *Tp53* knockout rats, which suggested that radiation-induced germ cell apoptosis is p53 dependent (25). Similarly, exposure to Ti3C2 nanosheets, a type of 2D nanomaterial, led to increased oxidative DNA damage and subsequent irreversible apoptosis mediated by the ATM/p53 signaling pathway in spermatogonia (58). Additionally, heat induced programmed necrosis in mouse testes is p53-dependent. Germ cell programmed necrosis was induced after heat shock at 42°C for 30 min, while *Tp53-/-* male mice were resistant to heat-induced necrosis (52).

Taken together, p53-dependent apoptosis and germ cell necroptosis are essential for maintaining appropriate sperm counts and ensuring sperm quality, which ensures successful spermatogenesis progression.

## 3 Signaling pathways of p53 in infertility

### 3.1 p53, oxidative stress and infertility

**Figure 2:**
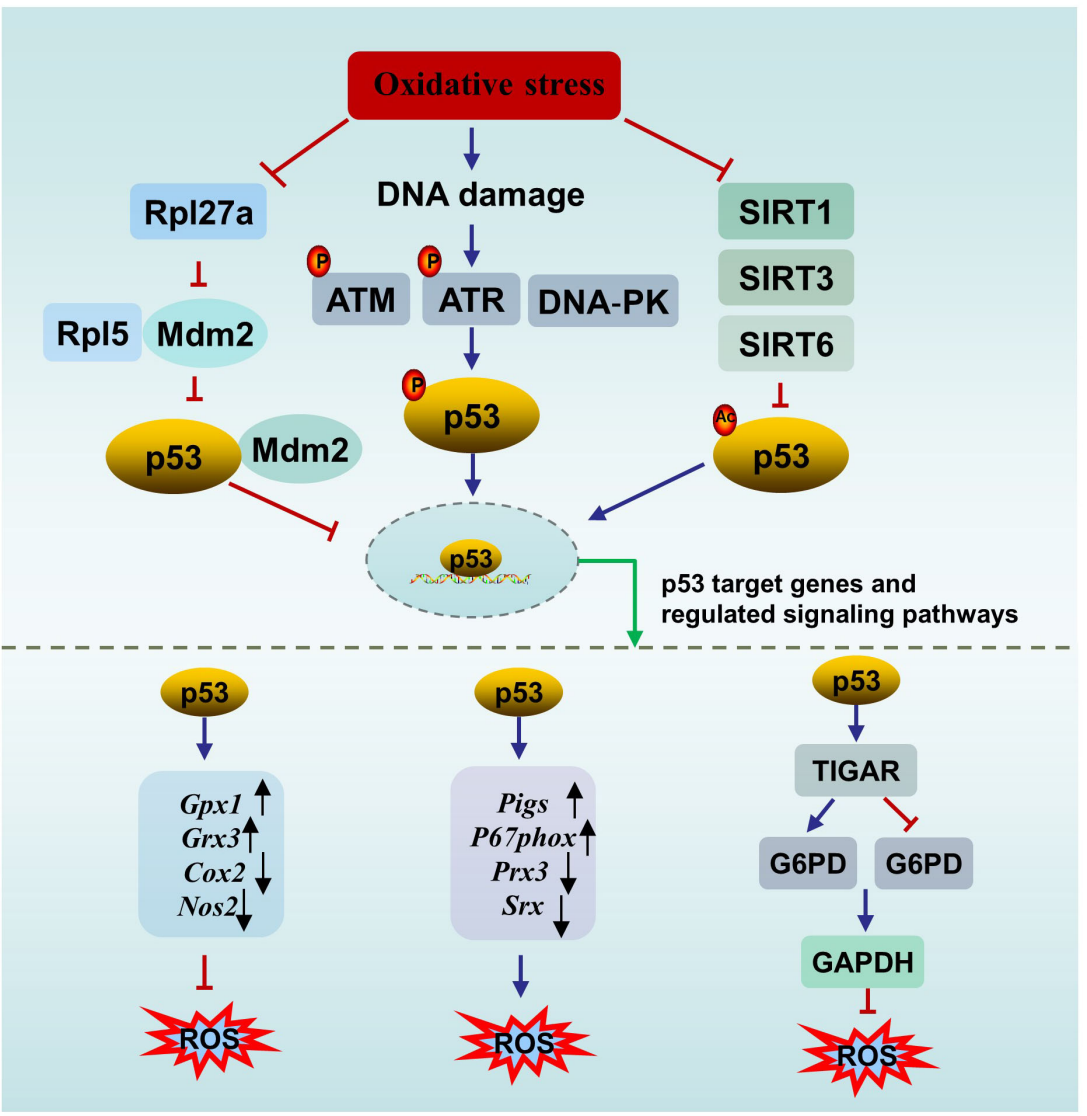
The interplay between p53 and oxidative stress. ATM, ataxia-telangiectasia mutated protein; ATR, ataxia telangiectasia mutated- and Rad3-related kinase; DNA-PK, the DNA-dependent protein kinase; SIRT, sirtuin; ROS, oxygen species; TIGAR, p53 upregulates the downstream gene TP53-induced glycolysis and apoptosis regulator; G6PD, glucose 6-phosphate dehydrogenase; NADPH, nicotinamide adenine dinucleotide phosphate.

Oxidative stress, which occurs when the antioxidant defense system fails to eliminate reactive oxygen species (ROS), is considered a primary cause of male infertility. A meta-analysis of 65 studies (3819 male infertility patients and 2012 controls) revealed elevated oxidative stress markers and decreased antioxidant defense in seminal plasma from patients with infertility (59). ROS are a kind of normal metabolic byproducts. An appropriate amount of ROS participates in several important physiological processes, including capacitation, hyperactivation and the acrosome reaction. Excessive ROS damage male reproduction via mechanisms related to lipid peroxidation, DNA damage, protein oxidation and mitochondrial dysfunction, leading to decreased reproductive capacity (60). p53 is involved in oxidative stress through several mechanisms (**Figure 2**).

First, p53 can directly affect oxidative stress or be affected by oxidative stress. On the one hand, p53 directly regulates redox signals by modulating multiple transcriptional targets associated with redox homeostasis and exerts bidirectional effects. p53 exerts prooxidant activity by upregulating its downstream targets, such as p53-inducible genes (*Pigs*) and the cytosolic subunit of the NADPH oxidase enzyme complex (*P67phox*), or repressing the expression of peroxiredoxin 3 (*Prx3*) and sulfiredoxin (*Srx*). While p53 exerts antioxidant effects by upregulating several antioxidant genes, such as glutathione peroxidase 1 (*Gpx1*) and glutaredoxin 3 (*Grx3*), or repressing the expression of dcyclooxygenase-2 (*Cox2*) and nitric oxide synthase 2 (*Nos2*) (61). In addition, under oxidative stimuli, p53 upregulates the downstream gene TP53-induced glycolysis and apoptosis regulator (TIGAR) to promote the conversion of the glycolysis pathway to the pentose phosphate pathway, thereby enhancing nicotinamide adenine dinucleotide phosphate (NADPH) production to counteract ROS. However, in an environment of excessive stimulation, persistent *Tigar* expression suppresses phosphofructokinase-1 activity and glucose 6-phosphate dehydrogenase (G6PD) activity, resulting in the shutdown of the glycolysis pathway without activating the pentose phosphate pathway, ultimately leading to testicular germ cell apoptosis and infertility (62, 63). The complex role of p53 in oxidative stress is not contradictory and is associated with the conditions of the cell. When the cell is in an uncontrollable state, p53 promotes oxidation to initiate programmed cell death. When the cell is in a normal state or a mild stress state, p53 induces antioxidant defense to maintain ROS at a low level to promote cell survival (64).

On the other hand, p53 is sensitive to redox conditions. This can be ascribed to 10 cysteine residues being easily oxidized by ROS (61). A reducing environment is required for the binding of p53 to DNA, in which the thioredoxin (Trx)/thioredoxin reductase (TrxR) system plays a vital role. The Trx/TrxR system is one of the major pathways involved in the regulation of redox homeostasis in male reproduction and can maintain the reduction of its substrates during sperm maturation (65). Ueno et al. (66) reported that Trx functions as a collaborator in p53 binding with DNA. A survey of 80 semen samples revealed that lower TrxR activity is accompanied by a reduction in the total antioxidant capacity (TAC) and in the number of thiol groups, which means that suppressed TrxR activity leads to a deteriorating oxidative status. In parallel, an obvious inverse correlation between p53 levels and TrxR activity was observed (67). In another study, Cordycepin (3’-deoxyadenosine), a nucleotide analogue from *Cordyceps militaris L.*, reversed the increase in p53 and Bax in aged rats by restoring antioxidant defense status through increasing the levels of enzymes (SOD, CAT, GPx, etc.) and nonenzymatic antioxidants (GSH, vitamin C, vitamin E, etc.), resulting in the amelioration of aging-mediated testicular impairment (68). All of these findings suggest an association between the activity of p53 and the redox state.

Second, p53 can be activated by DNA damage induced by ROS. For example, a study by Mu et al. (69) showed that *Dendrobium officinale* polysaccharides reduced p53-dependent apoptosis in mouse testicular cells induced by oxidative DNA damage, thereby ameliorating testicular injury and decreasing sperm quality. The decrease in the p53 level paralleled the degree of oxidative stress, and the degree of oxidative stress was dose-dependent (69). DNA damage mainly promotes p53 nuclear stability through three pathways to activate downstream targets (e.g., p21, p19, Bax, and Puma) to determine cell fate. Upon DNA DSBs, p53 is activated by the ATM/p53 pathway (70). Similarly, upon DNA single-strand breaks (SSBs), p53 is activated by the ataxia telangiectasia mutated- and Rad3-related kinase (ATR)/p53 pathway. In addition, the DNA‐dependent protein kinase (DNA‐PK) complex can phosphorylate p53 when the DNA strand breaks (71).

Third, p53 can be activated by oxidative stress-induced sirtuin (SIRT) suppression. There are seven types of nicotinamide adenine dinucleotide (NAD+)-dependent histone deacetylase enzymes in SIRTs, including SIRT1- SIRT7 (72). SIRTs can suppress the activity of p53 through posttranscriptional deacetylation of the p53 protein. SIRT1 in murine spermatogonia can be suppressed by oxidative stress of palmitic acid (PA) origin, resulting in elevated acetylated p53, which partly contributes to apoptotic cell death in spermatogonia and subfertility. Melatonin protects against PA-induced damage by restoring acetylated p53 to normal levels through the activation of the SIRT1/p53 pathway (73). Mir-34a is an oxidative stress-responsive RNA that is induced by oxidative stress (74). Mir-34a can directly bind to *Sirt1* mRNA, resulting in a decrease in the SIRT1 protein. It has been observed that acetylation of p53 increases via oxidative stress-induced activation of the miR-34a/SIRT1/p53 pathway, resulting in increased testicular cell apoptosis in a type 2 diabetic rat model (75). This observation is in accordance with the findings of Heydari et al. (76) in the testes of mice fed a high-fat diet. Similarly, SIRT3 can deacetylate and inactivate p53 in response to oxidative stress and DNA damage. Melatonin reversed PM2.5-induced GC-2 cell apoptosis through activating the SIRT3/p53 deacetylation pathway. The ameliorating effect of melatonin can be reversed by a specific inhibitor of SIRT3 (3-TYP) (71). Furthermore, it has been reported that SIRT6 is oxidized and suppressed under oxidative stress conditions (77). The BaZiBuShen formula, a Chinese herbal prescription, ameliorated sperm quality in D-galactose (D-gal)/NaNO2-induced aging mice by activating the SIRT6/p53 pathway via its antioxidant properties (78).

In addition, ionizing radiation triggers not only oxidative DNA damage but also ribosomal stress. Rpl5, a ribosomal protein, acts as both sensor and effector of ribosomal stress, which can bind to Mdm2 to inhibit its E3 ligase activity towards p53, thereby stabilizing p53 (79). Rpl27a, another ribosomal protein, may bind to both Mdm2 and Rpl5 to regulate p53 through Mdm2-Rpl5 complex. Under ribosomal stress, the expression of Rpl27a is down-regulated. Decreased Rpl27a weakens its ability to bind to Rpl5 and Mdm2 but promotes the binding of Rpl5 and Mdm2, resulting in the increased stability and activation of p53 and subsequent p53-dependent apoptosis, resulting in testicular structure abnormality and infertility (80, 81). Furthermore, the elimination of oxidative stress significantly inhibits p53-induced apoptosis (80).

In conclusion, p53 can directly regulate oxidative stress through downstream targets, and oxidative stress can subsequently affect p53 activity. Moreover, p53 can be activated by oxidative stress-induced DNA damage, SIRT suppression or ribosomal stress. These mechanisms link oxidative stress, p53 and impaired fertility together.

### 3.2 p53, apoptosis and infertility

The apoptosis of germ cells is essential for normal spermatogenesis, and p53 plays a key role in this process, as discussed in Section 2.3. Spontaneous apoptosis is characterized by relatively frequency in spermatocytes, a low frequency in spermatogonia, and infrequency in spermatids (67). However, deregulation of apoptosis in male germ cells is significantly related to male infertility (82). Under some pathological conditions and external stimuli, such as irradiation and toxicants, p53-dependent germ cell apoptosis increases dramatically, resulting in germ cell loss and infertility. For example, compared with those in normal controls, significantly greater expression of *Tp53* and *Casp3* and lower expression of the *Bcl-2* gene were detected in the testes of cyclopiazonic acid-exposed mice. Correspondingly, histomorphological abnormalities and decreased sperm numbers were observed in the testes of experimental mice (83).

**Figure 3:**
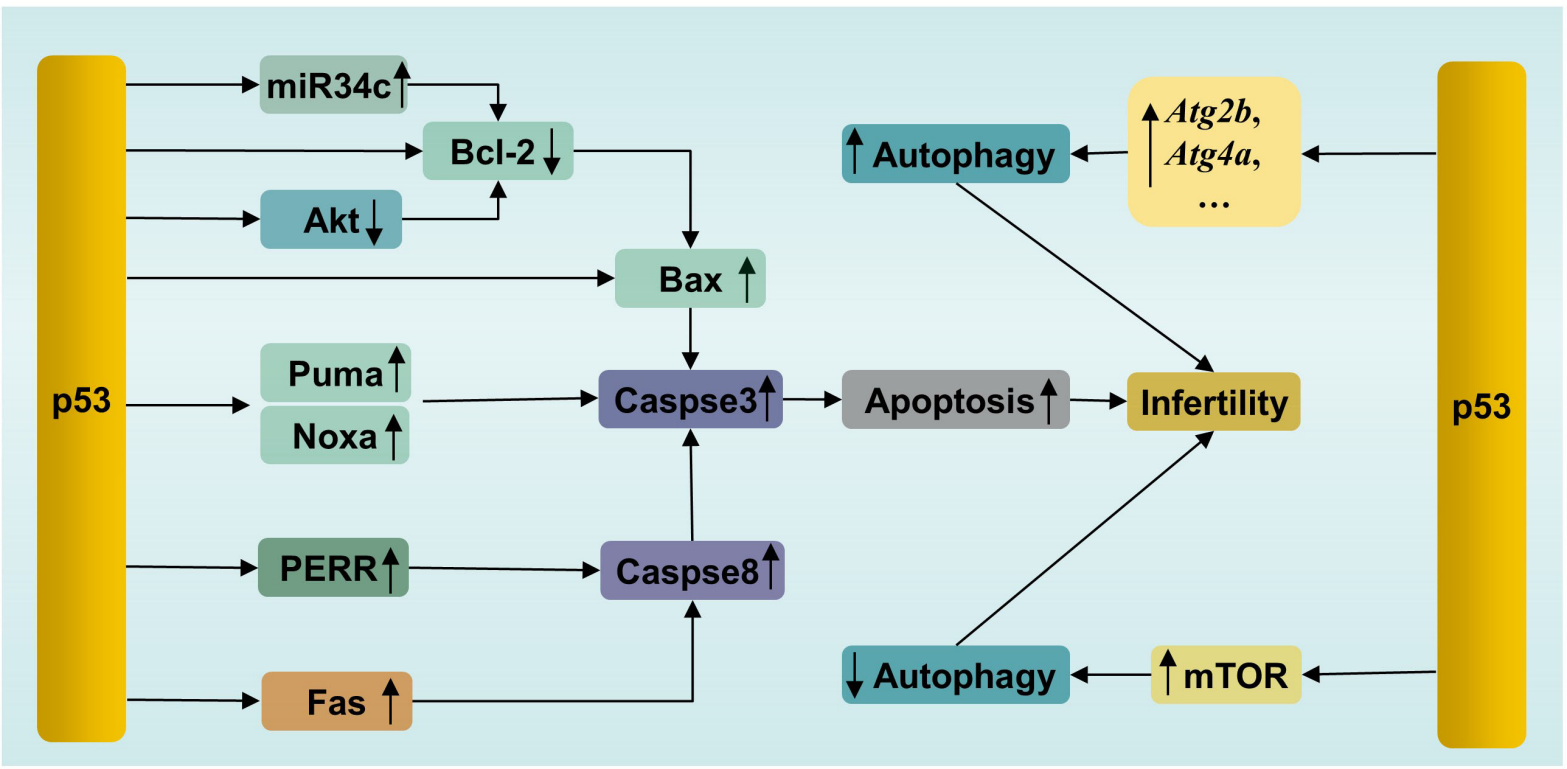
p53-mediated apoptosis and autophagy in male infertility. PERR, p53 apoptosis effector related to PMP-22; Fas, factor associated with suicide.

p53 triggers apoptosis in male germ cells through several pathways (**Figure 3**). (1) Activated p53 induces apoptosis by promoting the transcription of downstream proapoptotic genes, including *Puma*, *Bax* and *Noxa*. (2) p53 triggers transcription-independent apoptosis by binding directly to antiapoptotic Bcl-2 at the site of the BH3 binding pocket. The binding sites of p53 in Bcl-2 are the same as those of Bax for binding to Bcl-2, leading to the release of Bax and activation of apoptosis (84). (3) p53 can induce apoptosis by promoting the expression of the downstream transcriptional target *Perp* (p53 apoptosis effector related to PMP-22). It has been reported that *Mkrn2* (E3 ubiquitin-protein ligase makorin-2) gene KO in mice leads to impaired spermatogenesis mediated by p53/PERP-mediated apoptosis (85). In parallel, a greater level of *PERP* expression has been detected in the semen of patients with oligoasthenoteratozoospermia (OAT) than in that of normal individuals (85). PERP stimulates apoptosis by activating Caspase-8 to trigger the mitochondrion-Cytochrome c-caspase protease pathway or facilitating extracellular calcium loading into the endoplasmic reticulum (86). Interestingly, there is a positive feedback loop between PERP and p53. PERP promotes p53 phosphorylation and hinders Mdm2-mediated ubiquitination-mediated degradation of p53, leading to the subsequent stability and activation of p53 (87). (4) p53 drives miR34c expression to promote apoptosis by inhibiting the activity of the antiapoptotic genes *Bcl-2* or *c-Myc* (88). Like other miRNAs, miR34c can inhibit the transcription or initiate the degradation of target gene mRNAs by binding to the 3′UTR (89). A study showed significantly increased levels of miR-34c and p53 in semen from patients with nonobstructive azoospermia and moderate OAT (90). (5) Testicular cell apoptosis can be mediated by the p53-associated extrinsic factor associated with suicide (Fas)/Fas ligand (FasL) death signaling pathway (91). In addition, activated p53 inactivates the serine/threonine kinase Akt by promoting Akt proteolysis mediated by caspase, or by transactivating the *PTEN* gene, which encodes a phosphatidylinositide phosphatase to prevent Akt activation (92). Subsequently, inactivated Akt depresses downstream nuclear factor Bcl-2 expression to initiate apoptosis (93). Besides, proapoptotic protein Bad, a downstream apoptosis promoter in p53-induced apoptosis in the testis, is inactivated by Akt, resulting in decreased apoptosis (94, 95). Thus, inactivated Akt results in increased apoptosis in testis.

In short, p53 can induce excessive apoptosis via the intrinsic and extrinsic apoptotic pathways to impair male fertility (96).

### 3.3 p53, autophagy and infertility

Autophagy is a conserved lysosome-dependent biological process responsible for cellular degradation and recycling, including cytosolic proteins and damaged organelles. Once autophagy is triggered, phagophores are formed first, starting with activation of the UNC-like autophagy-activating kinase 1 (ULK1) initiation complex, followed by autophagy-related protein (ATG)-driven autophagosome formation, in which autophagic cargo is segregated. Subsequently, autophagosomes fuse with lysosomes, where cargo is degraded (97). Under physiological conditions, autophagy participates in multiple physiological processes during spermatogenesis, including spermatid polarization, cellular remodeling and maintenance of the acrosome (98). However, autophagy has two effects on cells. Briefly, moderate induction of autophagy plays a cytoprotective role, which promotes cell survival by removing damaged cell components. However, excessive autophagy causes autophagic death (99). This feature of autophagy is no exception for male germ cells. For example, suppressed autophagy has been observed in flutamide-induced neonatal cryptorchid infertile rats, and retinoic acid improved spermatogenesis by activating autophagy via inhibition of the phosphatidylinositol 3 kinase (PI3K)/Akt/mammalian target of rapamycin (mTOR) signaling pathway (100). In contrast, in another study, excessive autophagy and increased apoptosis have been detected in a murine model of oligoasthenospermia induced by treatment with cyclophosphamide. Schisandrin A, a helpful bioactive lignan, improved sperm quality by mitigating excessive autophagy-induced apoptosis by promoting Akt phosphorylation in this study (101). Akt is one of regulatory proteins connecting apoptosis and autophagy (102). Phosphorylated Akt, on one hand, inhibits autophagy by activating the Akt/mTOR pathway, on the other hand, inhibits apoptosis by phosphorylating Mdm2 at Ser 166 to facilitate the degradation of p53 (103). These studies confirmed that dysregulation of autophagy is related to infertility, as well as the interplay between apoptosis and autophagy.

p53, similar to Akt, is involved not only in apoptosis, as mentioned above, but also in the regulation of autophagy (**Figure 3**). p53 plays dual roles in autophagy in male germ cells. On the one hand, activated p53 can enhance autophagy by directly inducing the expression of autophagy genes. p53-dependent induction of autophagy genes has been observed in mouse and human cells under DNA damage conditions (104). Many autophagy genes have been identified as p53 target genes (e.g., *Atg2b*, *Atg4a*, *Atg7*, *Atg10*, and *Ulk2*) (104). It has been confirmed that p53 promotes autophagy under various stimuli, such as DNA damage and genetic activation (104). Microcystin-leucine-arginine (MC-LR), an intracellular toxin, can trigger oxidative stress-mediated DNA damage, leading to the presence of DSBs, which can be recognized by ATM. Subsequently, ATM phosphorylates the downstream protein p53 and activates p53-induced autophagy by increasing the levels of autophagy proteins, such as Beclin1, ATG5 and LC3-I, resulting in pathological damage in murine testes. In parallel, treatment with the ATM inhibitor KU55933 suppressed autophagy and alleviated this damage by inhibiting the ATM/p53 pathway (105). On the other hand, p53 suppresses autophagy in some cases. *In vitro* experiments showed that busulfan inhibited autophagy by phosphorylating Akt at Ser473 and subsequently activating the Akt/mTOR pathway, resulting in massive SPCs death. Interestingly, an increase in the level of phosphorylated p53 was accompanied by mTOR-mediated autophagy suppression after busulfan treatment for 2 h to 8 h. Moreover, p53-deficient SPCs exhibited attenuated autophagy inhibition and decreased p-mTOR after busulfan treatment for 6 h. In general, p53 participates in busulfan-induced autophagy inhibition by partly activating mTOR in SPCs (106). The mechanisms by which p53 activates mTOR have been clearly clarified in a recent review by Cui et al. (107). The bilateral effects of p53 on autophagy are considered to be related to its intracellular localization. The nuclear localization of p53 induces autophagy, while the accumulation of p53 in the cytoplasm inhibits autophagy (108). Notably, a previous study demonstrated that p53 induced SPC apoptosis under busulfan stress (70). This suggested that p53 participated in the fine regulation of apoptosis and autophagy in male germ cells. It is still unclear how p53 initiates apoptosis or autophagy under the same stress conditions. The selection tendency of p53 may be related to the intensity and duration of stress, which enable p53 to execute cellular responses suitable for specific cellular states.

## 4 p53 and male infertility-related diseases

The indispensable role of p53 in spermatogenesis suggests that p53 is closely associated with male infertility and various infertility diseases. The activation of p53 was observed in testicular samples from infertile patients (109). Here, we showed several male infertility-related diseases associated with p53.

### 4.1 OAT

OAT includes oligospermia and asthenozoospermia, which refers to a sperm cell count lower than 15 million/ml in male ejaculate and/or total motility <40% and progressive motility <32% in a semen sample, and abnormal sperm morphology (110, 111). Several studies have indicated that p53 is elevated in oligoasthenozoospermia. Compared to that in the normozoospermic group, the level of p53 in semen samples from asthenozoospermic donors is greater, as is the level of ROS, leading to increased levels of apoptotic sperm in the ejaculatory semen (67). Another study involving 29 men (11 with asthenozoospermia and 18 controls) showed that the expression of p53 is greater in asthenozoospermic men and that there is a significant negative correlation between p53 levels and sperm motility, as well as between p53 levels and sperm concentration (112). Similarly, a study by Rahbar et al. (113) showed that sperm and testicular tissue samples from individuals with severe OAT and moderate OAT had significantly greater expression of the *TP53* and *CASP9* genes than those from individuals in the normal sperm group. In parallel, a significant association between p53 and miR‐15b, a microRNA that can stimulate p53 phosphorylation, was also observed in this study (113). Briefly, aberrant activation of p53 might contribute to OAT.

### 4.2 Varicocele

Varicocele, defined as an abnormal expansion of the pampiniform plexus of gonadal veins above the testicle, is one of the most common causes of male infertility (114). Increased expression of p53 and BAK was observed in ejaculated semen samples from varicocele patients, suggesting the occurrence of remarkably active p53-dependent apoptosis (115). Spermatocytes of the testis are considered the cells mainly affected by varicocele-associated apoptosis (115). The increase in p53, Cytochrome c, and poly(ADP-ribose) polymerase (PARP)-1 levels has also been observed in varicocele-induced rats, which indicated that oxidative stress-induced p53 activation plays a role in varicocele (116). Moreover, another study showed elevated levels of phosphorylated p53 and a marker of DBSs, phosphorylated histone γ-H2AX accumulation in a time course-dependent manner in varicocele murine models, suggesting that activation of p53-dependent apoptosis is mediated by γ-H2AX (117). Furthermore, an inverse association has been observed between the proportion of the *TP53* codon 72 Arg/Arg genotype, and sperm motility in men with varicocele (118). Codon 72 has a polymorphism that encodes the amino acids arginine (Arg 72) or proline (Pro 72), which influences the structure and function of p53. The Arg 72 variant of p53 more potently induces apoptosis by enhancing its interaction with the mitochondria and promoting the release of cytochrome c release (119). The increased testicular temperature has been observed in experimental varicocele models and in man, and is widely considered to be a mechanism by which varicocele affects testicular function. And p53 acts as a master regulator in germ cell apoptosis induced by hyperthermia (120, 121). Those findings suggest that p53 is associated with infertility caused by varicocele.

### 4.3 Cryptorchidism

Cryptorchidism, a condition in which one or both testes fail to descend into the scrotum, is associated with an increased risk of infertility (122). A rise in testicular temperature was considered a main factor for the impairement of testicular functions in experimental induced cryptorchidism in rodents or men with cryptorchidism (123, 124). A study revealed that the initial phase of germ cell apoptosis induced by heat stress caused by experimental cryptorchidism was mediated by p53-dependent pathway (125). Another study by Zhou et al. (126) showed increased levels of p53 and Fas protein, as well as testicular cell apoptosis, and decreased testicular weight in a surgery-induced cryptorchidism murine model. Moreover, a reduction in the levels of p53 and Fas mediated by the overexpression of *Cirp* (cold-inducible RNA-binding protein) ameliorated the increase in germ cell apoptosis and testicular injury induced by cryptorchidism. Additionally, testicular orphan receptor-2 (TR2), a member of the steroid-sensitive hormone receptor superfamily is involved in p53-mediated cryptorchidism-related infertility. TR2 is highly expressed in testes, and can regulate retinoic acid receptors/retinoid X receptors (RARs/RXRs) signal pathways (127, 128). A study showed that the expression level of TR2 mRNA decreased with the progression of p53-mediated germ cell apoptosis in the cryptorchid testis of rat and rhesus monkey (128). Future research showed that TR2 was repressed by activating the p53/retinoblastoma gene product (Rb) signaling pathway through the direct combination of Rb and TR2 in surgery-induced cryptorchid testes of rhesus monkeys (129). These results suggest that p53 is involved in cryptorchidism-induced infertility.

### 4.4 Idiopathic male infertility

Idiopathic male infertility refers to infertility in men with abnormal sperm parameters after excluding possible factors and is responsible for approximately 40% of cases (130). Several studies have shown that p53 polymorphisms are associated with idiopathic male infertility. For instance, a study conducted by Mashayekhi et al. (131) revealed a more frequent Arg allele of the *TP53* codon 72 polymorphism in men with idiopathic infertility than in controls. In addition, a case-control study including 580 infertile patients and 580 controls showed a significantly increased risk of genetic variants in *TP53* (rs2287498) and *MDM2* (rs937283) associated with idiopathic male infertility in a Chinese population (132). However, inconsistent conclusions exist. A case–;control study including 198 idiopathic infertile patients and 233 fertile controls revealed no association between the codon 72 and IVS7 + 72C>T polymorphisms of the *TP53* gene and spermatogenetic failure (133). Similarly, another investigation conducted by de Morais et al. (134) revealed no association between the *TP53* polymorphism in codon 72 and male idiopathic infertility. It seems that whether the *TP53* polymorphism is involved in the pathogenesis of idiopathic male infertility is associated with different demographic factors.

### 4.5 Testicular torsion

Testicular torsion is a urological emergency accompanied by scrotal pain and ischemia and subsequent testicular ischemia–;reperfusion injury (tIRI), resulting in cell damage and even infertility (135, 136). A study by Shamsi-Gamchi et al. (137) showed that testicular torsion caused severe DNA damage induced by oxidative stress, leading to markedly increased expression of *Tp53*, *p21*, *cyclin D1*, *Bax* and *Casp3* in a time-dependent manner, as well as a significant increase in the apoptosis index, resulting in deteriorated testicular tissue and decreased sperm count in murine testicular tissue within 8 hours after testicular torsion. Mechanically, p53 induced by DNA damage activates p21. Subsequently, p21 binds with Cdk4 to suppress the Cyclin D1/Cdk4-related cell cycling process and finally induce p53/p21-dependent apoptosis (137). Similarly, another study showed that tIRI triggered oxidative stress, thereby upregulating the phosphorylation of p53 and the expression of its downstream proapoptotic factor Puma, resulting in increased apoptosis. In addition, during tIRI, p53 induces sustained expression of the gene *Tigar*, leading to the failure to convert glycolysis to the pentose phosphate pathway, resulting in testicular germ cell apoptosis (62, 63). In summary, p53-dependent apoptosis and cell cycle arrest are related to testicular injury caused by testicular torsion.

## 5 Potential therapeutic agents affecting the p53-related pathways in male infertility

Recently, efforts have been made to develop p53-targeted therapies for certain diseases, such as cancer and neuroinflammation (138, 139). As a result, many potential therapeutic agents, including natural and synthetic drugs and small molecule compounds, have been developed. The effects of several agents affecting the p53-related pathways on male infertility are shown in **Table 2**. Among them, pifithrin-α, a p53 specific inhibitor, directly targets p53 by obstructing the p53 protein in the nucleus (144). Excitingly, innovative therapeutic strategies targeting p53, including Mdm2 inhibitors and mutant p53-restoring compounds, have shown great potential in cancer treatment in clinical trials (145). It can be expected that innovative approaches targeting p53 can be implemented for the treatment of male infertility in the near future.

**Table 2:** Agents for treating male infertility via the p53-related pathways.

Agent	Mechanism	Study model	Effect	Ref.
Pifithrin-α	Inhibit p53-Sox3 signaling pathway	*Csnk1a1* conditional knockout mice	Increase *Sox3+* cell numbers	(34)
Melatonin	Activate Sirt3/p53 pathway	ApoE^-/-^ mice; GC-2 cells	Inhibit apoptosis	(46)
miRNA‐138‐5p	Inhibit p53/Bax/Bcl-2 pathway	1. SD rats exposing to cigarette smoke; 2. Leydig and Sertoli cells under cigarette smoking-treated	1. Attenuate testicular injury 2. Suppress cell apoptosis	(140)
Dapagliflozin	Reverse the overexpression of *Tp53*	Streptozotocin-induced diabetes mice model	Attenuate the frequency of disomic and diploid sperm	(141)
Resveratrol	Downregulate the expression of p53 and Bax; upregulate the expression of Bcl-2	Cadmium chloride-induced testicular damage marine models	Inhibit apoptosis	(142)
Ghrelin	Activation of p53-dependent DNA repair mechanisms	Cisplatin-induced murine testicular damage	1. Attenuate testicular weight 2. Increase epididymal sperm count 3. Improve sperm motility	(143)

## 6 Conclusion remarks

p53 plays a central role in the response to cellular stress and is involved in multiple biological processes. Herein, we present an overview of the essential roles of p53 in normal spermatogenesis and the possible mechanisms by which p53 is involved in male infertility. We conclude that mutated p53 is a risk factor for male infertility and aberrant p53 expression might be related to male infertility. Recently, considerable efforts have been made to develop small molecules targeting p53, which is highly promising for cancer therapies (146). Although testicular microenvironment and pathology differ from the tumoral, considering the high potential of p53 to be targeted for drug therapy, insights into the interplay between p53 and spermatogenesis and male infertility are needed, which opens up avenues for novel p53-based therapeutic interventions. In the context of male infertility, combined therapies that both alleviate testicular stress and target p53 and/or its activated pathways should be more effective than focusing on one alone. However, several questions remain to be answered. How does p53 balance apoptosis and autophagy? Clarification of the specific mechanism that determines this effect is urgently needed. Spermatogenesis is a unique and complex cell cycle process required for fine regulation. The level of p53 must also be precisely regulated. Therefore, when targeting p53, which indicator can be used to detect whether the activation or inhibition of p53 is appropriate? Addressing these concerns will aid in the clinical treatment of infertility with strategies based on the attractive target p53.
